# Translational insights from nonclinical studies of AAV gene therapies for hemophilia: mechanisms underpinning variability and durability of gene expression

**DOI:** 10.1177/20406207251406537

**Published:** 2026-01-29

**Authors:** Sylvia Fong, Laura L. Swystun, Paul Batty, David Lillicrap

**Affiliations:** Department of Pathology and Molecular Medicine, Queen’s University, Kingston, ON, K7L 3N6, Canada; Department of Pathology and Molecular Medicine, Queen’s University, Kingston, ON, Canada; Department of Pathology and Molecular Medicine, Queen’s University, Kingston, ON, Canada; Department of Haematology, Cancer Institute, University College London, London, UK; Department of Pathology and Molecular Medicine, Queen’s University, Kingston, ON, Canada

**Keywords:** adeno-associated virus, animal models, durability, genetic therapy, hemophilia, human liver biopsy, mechanisms of action, models, variability

## Abstract

Adeno-associated virus (AAV) gene therapy is a promising approach for hemophilia, offering the potential for sustained therapeutic expression of coagulation factors. However, both variability and durability of transgene expression remain a challenge, limiting treatment predictability. Comparative preclinical and human liver biopsy studies suggest that transcriptional efficiency, rather than vector genome copy number (VCN), is a primary determinant of variability and durability in treatment response. Despite the presence of vector genomes in hepatocytes, transcriptional output varies significantly across species and individuals, indicating that VCN alone is insufficient to predict therapeutic efficacy. This review synthesizes findings from preclinical models (mice, dogs, non-human primates (NHPs), and human hepatocytes) and clinical liver biopsy studies to examine mechanisms influencing AAV gene therapy variability and durability. While vector genome retention is relatively comparable across species, transcriptional efficiency declines in higher species, particularly in NHPs, dogs, and humans. Beyond transcription, vector genome loss, hepatocyte turnover, immune responses, and cellular stress (e.g., endoplasmic reticulum (ER) stress) may contribute to intraindividual declines in transgene expression over time. Recent findings also highlight the role of epigenetic modifications, vector integration patterns, and translational shutdown linked to protein-folding stress in influencing durability. Expression patterns show greater long-term stability with factor IX (FIX) gene therapy compared to factor VIII (FVIII), which often declines more sharply. Distinctions may reflect differences in protein biosynthetic burden and cellular stress responses, particularly for FVIII. Most FIX trials use the highly active Padua variant, enabling lower expression levels with potentially less cellular stress, while the tendency of FVIII to misfold and trigger ER stress may contribute to transcriptional or translational shutdown over time. Integrating insights from preclinical models, human liver biopsies, and ongoing clinical trials, this review refines our understanding of AAV gene therapy variability and durability, ultimately guiding next-generation gene therapies to enhance long-term clinical efficacy.

## Plain language summary

Animal models of AAV gene therapy in hemophilia

Adeno-associated virus (AAV) based gene therapy is a promising new treatment for hemophilia. It works by using AAV as a delivery vehicle (vector) to carry the gene for the missing blood clotting protein, either factor VIII or factor IX, into the liver. Liver cells then use this genetic information to produce the clotting protein, which is released into the bloodstream. Gene therapy does not work the same for everyone. Some people make more clotting protein than others (variability), and in some, the effect lasts longer than in others (durability). Carefully conducted studies in animal models of hemophilia, as well as analyses of human liver biopsy samples after gene therapy, have helped us understand the reasons for these differences. Even when a similar amount of genetic material is delivered to the liver, the body’s ability to use it to make protein, a process involving transcription and translation, can vary. Several factors may influence how well and how long gene therapy works, including how the AAV vector enters the liver cell, the loss or turnover of liver cells, loss or suppression of AAV vectors over time, natural immune responses, and cellular stress. In this review, we summarize key insights gained from animal models, laboratory studies, and ongoing clinical trials to improve our understanding of how AAV gene therapy works for hemophilia.

## Introduction

The cloning of the *F8* and *F9* genes 40 years ago^1,2^ was accompanied by predictions of the translational benefits that could derive from this new knowledge.^3^ In the intervening decades, these predictions have generally been proven to be correct. Pathogenic mechanisms resulting in hemophilia are now more widely understood, molecular diagnostic testing is integrated into routine clinical care, and recombinant bioengineered protein therapeutics are used worldwide. The one prediction that remains only partially realized is the development of gene therapy for these disorders.

Between 1995 and 2010 a series of small-scale phase I-II clinical studies were completed utilizing adenoviral, retroviral, and adeno-associated virus (AAV) vectors, but it wasn’t until 2011 that persistent low expression of factor IX (FIX) was documented in a small cohort of AAV-treated severe hemophilia B patients.^4^ Now, in 2025, 464 hemophilia A and B patients have received AAV-mediated gene therapy,^5^ and in 2022 the first regulatory approvals were issued for both factor VIII (FVIII) and FIX gene therapy products.^6^

Throughout the development of these therapies, preclinical studies in hemophilic mice, dogs, and nonhuman primates (NHPs), in addition to cell models have played important roles in assessing treatment safety and efficacy.^7,8^ In particular, these studies have enabled more controlled and detailed investigation than would be possible in human patients, and in general, the findings in animal models have been predictive of outcomes in the clinic.^9,10^

## AAV gene therapy for hemophilia: Mechanism of action and current landscape

### Overview of AAV vector platforms

AAV are small, replication-defective, nonenveloped dependoparvoviruses with a 4.7 kb single-stranded DNA genome. Recombinant AAV (rAAV) gene therapy vectors consist of a transgene, promoter/enhancer elements flanked by AAV2-genome-derived inverted terminal repeat (ITR) regions contained within an icosahedral capsid.^11,12^ AAV vectors are produced using mammalian cells, such as HeLa cells, human embryonic kidney (HEK293) cells, or baculovirus-transfected insect cells such as *Spodoptera frugiperda* (Sf).

For hemophilia B gene therapy, the *F9* cDNA is 1.6 kb in length and can be packaged by AAV without modification or truncation of the transgene cassette. All recent hemophilia B gene therapies use the FIX Padua (FIX-R338L) gain-of-function variant that increases FIX specific activity eightfold compared with wild-type (wt) FIX.^13–16^ For hemophilia A gene therapy, B-domain deleted *F8* cDNA is used to overcome packaging capacity limitations of AAV vectors. Most AAV FVIII gene therapies use the FVIII-SQ variant, the same form used in recombinant FVIII products. One study has employed the FVIII-V3 variant, which includes a truncated B-domain spacer retaining six *N*-glycosylation sites to improve FVIII secretion.^17–19^

The AAV serotype is categorized based on variations in the structure of its capsid proteins, which determine its tissue tropism.^20^ Hemophilia gene therapies use a number of naturally occurring AAV capsid serotypes as well as bioengineered proprietary variants. An overview of currently approved and investigational hemophilia AAV gene therapies is provided in Tables 1 and 2, updated from Pierce et al.^17^

**Table 1.** Current approved and investigational FVIII gene therapies.

Therapy	Sponsor	Capsid	Transgene	Promoter	Platform	Trial #	Phase Dose	Dose (vg/kg)	Number of participants	Current status	References
Valoctocogene roxaparvovec (Roctavian, AAV5-FVIII-SQ)	BioMarin Pharmaceutical Inc.	AAV5	BDD hFVIII-SQ	HLP	Sf9	NCT03370913 (GENEr8-1)	III	6 × 10^13^	132	Approved in the United States, conditionally approved in Europe	21,22
						NCT02576795	I/II	4 × 10^13^ to 6 × 10^13^	7		24–26
Giroctocogene fitelparvovec (PF-07055480, SB-525)	Sangamo/Pfizer	AAV6	BDD-FVIII	Minimal transthyretin promoter	Sf9	NCT04370054 (AFFINE)	III	3 × 10^13^	50	Clinical development discontinued	27
						NCT03061201 (Alta)	I/II	9 × 10^11^ to 3 × 10^13^	11		23, 28
Dirloctocogene samoparvovec (SPK-8011)	Spark/Roche	AAV-LK03 (Spark200)	BDD-FVIII	Liver-specific promoter	HEK 293	NCT03003533 and NCT03432520	I/II	2 × 10^12^	9	Clinical development discontinued	29
Peboctocogene camaparvovec (BAY 2599023, DTX-201)	Bayer/Ultragenyx	AAVhu37	BDD-FVIIIco	Liver-specific promoter/enhancer	HeLa	NCT03588299	I/II	6 × 10^11^	9	Active	30, 31
AAV-HLP-hFVIII-V3	University College London/St Jude Children’s Medical Center	AAV8	hFVIII-V3	HLP	HEK 293	NCT03001830 (GO-8)	I/II	6 × 10^11^ to 6 × 10^12^	12	Active, not recruiting	18, 32
GS001	Chinese Academy of Medical Science, Institute of Hematology and Blood Diseases Hospital	AAV8	BDD-FVIII	Liver-specific promoter	N/A	NCT04728841	I/II	2 × 10^12^ to 4 × 10^12^	12	Active	33

Manufacturer’s nomenclature is used in the “Transgene” column. All *F8* transgenes are B-domain deleted and codon-optimized; all use the SQ variant except AAV-HLP-hFVIII-V3.

AAV, adeno-associated virus; BDD, B-domain deleted; CO, codon-optimized; HEK, human embryonic kidney; HLP, hybrid liver*-*specific promoter; N/A, not available.

**Table 2.** Current approved and investigational FIX gene therapies.

Therapy	Sponsor	Capsid	Transgene	Promoter	Platform	Trial #	Phase	Dose (vg/kg)	Number of participants	Current status	References
Etranacogene dezaparvovec (Hemgenix, AMT-061)	UniQure/CSL	AAV5	FIX Padua, CpG-reduced cassette	LP1	Sf9	NCT03569891 (HOPE-B)	III	2 × 10^13^	54	Approved in United States and Canada, conditionally approved in Europe	14, 34, 35
							IIb	2 × 10^13^			36, 37
Fidanacogene elaparvovec (Beqvez, SPK-9001/PF-06838435)	Spark/Pfizer	AAV rh74	FIX Padua	Liver-specific promoter	HEK 293	NCT03861273 (BENEGENE-2)	III	5 × 10^11^	45	Approved in United States and subsequently discontinued	15
						NCT02484092, NCT03307980	I/IIa	5 × 10^11^	15		38, 39
Verbrinacogene setparvovec (FLT180a)	Freeline	AAVS3	FIX Padua	FRE1 (liver-specific promoter)	HEK 293	NCT03369444 NCT03641703 (B-AMAZE)	I/II	3.84 × 10^11^ to 1.28 × 10^12^	10	Clinical development discontinued	40, 41
CSL220 (AMT-060)	UniQure	AAV5	hFIXco, CpG-reduced cassette	LP1	Sf9	NCT02396342	I/II	5 × 10^12^to 2 × 10^13^	5	Active	42,43
scAAV 2/8-LP1-hFIXco	University College London/St Jude Children’s Medical Center	AAV8	hFIXco, CpG-reduced cassette	LP1	HEK 293	NCT00979238	I/II	2 × 10^11^ to 2 × 10^12^	6	Active	44, 45
BBM-H901	Chinese Academy of Medical Science, Institute of Hematology and Blood Diseases Hospital	AAV843, gene shuffled liver-tropic capsid	FIX Padua, CpG-motif-free	LXP2.1	HEK 293	NCT05203679	III	5 × 10^12^	26	Active	46
						NCT04135300	I	5 × 10^12^	10		13, 47

Notably, several gene therapy constructs incorporate codon optimization to enhance transgene expression. While codon optimization is generally beneficial, such strategies may also increase CpG content and trigger innate immune response via TLR9 that could impact long-term durability.^48,49^

AAV, adeno-associated virus; CO, codon-optimized; HEK, human embryonic kidney; N/A, not available.

### Cellular and molecular mechanisms of AAV gene therapy

The pathway(s) by which AAV gains entry to and traverses through a target cell are of importance for understanding the variability in response observed between vectors and intraindividual variability for individuals treated with the same vector construct. This topic has been the subject of a number of detailed recent reviews summarized in brief in this section.^50–53^ Each step can act as a bottleneck regulated by host cell factors and vector components. Additionally, preexisting immunity to AAV, particularly neutralizing antibodies and memory T-cell responses, can contribute to interindividual variability by limiting vector delivery and persistence. Of note, much of these data are derived from in vitro studies as these processes are difficult to assess experimentally in vivo.

The first step is cellular internalization of AAV, with organ and cellular specificity largely defined by the AAV capsid. Internalization requires initial cell surface interactions with glycans, including heparan sulfate proteoglycan (AAV2, AAV3B, AAV6, and AAV13),^54^ O-linked α2-3 sialic acid (AAV4), α2-3 and α2-6 *N*-linked sialic acid (AAV1, AAV5, and AAV6), and N-linked galactose (AAV9). Given the low specificity of these interactions, it is now believed these glycans facilitate the accumulation and orientation of AAV at the cell surface rather than functioning as primary receptors.^50^ Additional proteinaceous (co)receptors have been described including CD9, epidermal growth factor receptor, fibroblast growth factor receptor 1, hepatocyte growth factor receptor (HGFR/MET), αVβ5 and α5β1 integrin, laminin receptor, and α and β platelet-derived growth factor (PDGFR), which bind different capsid serotypes.^55^ The AAV receptor (AAVR) is a required entry factor for successful transduction of multiple AAV capsids, with notable exceptions being AAV4 and AAVrh32.33.^50,55^

Cellular intake occurs via different pathways, including clathrin-mediated endocytosis, clathrin-independent carriers, and GPI-enriched endocytic compartment endocytosis and micropinocytosis. Intracellular pathways of AAV are less well defined and predominantly based on in vitro data. Cellular trafficking involves the microtubule network,^56^ with transit through endosomal pathways and retrograde transport to the trans-Golgi network (TGN), a process that is dependent on Syntaxin 5.^57 [Bibr bibr58-20406207251406537]^ The capsid undergoes conformational change in the increasingly acidic endosomal compartments resulting in exposure of a phospholipase A2 (PLA2) domain in the unique N-terminus of the VP1 (VP1u) capsid protein. This process is important to allow endosomal escape, which then leads to accumulation of AAV in the perinuclear space. Recent data has proposed a role for a second AAV entry factor, the G-protein coupled receptor 108 (GPR108) in endosomal escape at the TGN for all capsids except AAV5.^58^ Interestingly, earlier studies using fluorescently labeled rAAV in HeLA cells supported different rates of cellular entry and intracellular trafficking to the nucleus, depending on the capsid serotype. In this study, AAV2 demonstrated slower rates of uptake, intracellular trafficking, and nuclear import compared to AAV5 and AAV1.^59^

The next rate-limiting stage involves import into the nucleus through the nuclear pore complex (NPC). This process requires interaction between a nuclear localization signal (externalized VP1u) and members of the importin protein receptor family (IMPα and IMPβ1). A second NPC-dependent pathway of AAV nuclear entry via transient permeabilization of the nuclear envelope has also been proposed.^60,61^ AAV dissociates from the importin complex in the nucleoplasm via RanGTP^61^ and transits through the nucleolus where capsid uncoating is thought to occur. This results in the delivery of a single-stranded DNA genome. Formation of a transcriptionally active double-stranded structure can occur via second-strand synthesis or through annealing of complementary genomes followed by DNA repair; both mechanisms are thought to contribute, with their relative importance still under investigation. In nondividing cells such as hepatocytes, DNA repair pathways may play a particularly critical role, given limited DNA synthesis activity. The endonuclease Artemis facilitates resolution of AAV ITRs, supporting efficient vector processing.^62^ Transcription of the AAV genome involves host factors such as the splicing regulator PHF5A (transcriptional elongation by RNA polymerase II),^63^ the E3 ubiquitin ligase RNF121 (a pan regulator of AAV transcription),^64^ and the liver-specific transcription factor HNF1α.

In vitro and in vivo studies have characterized mechanisms of AAV persistence. For wild-type AAV, evaluation of human tissue samples has demonstrated that episomal forms were the predominant mechanism of persistence in vivo.^65^ Despite this finding, integration for wt-AAV2 has been well-characterized as an alternative mechanism of vector persistence. This process of integration is site-specific and directed by the Rep78/68 complex at Rep binding sites (RBS) in the host genome. These findings led to the characterization of a recurrent integration site in vitro in HeLa cells on chromosome 19 in exon 1 of the phosphatase 1 regulatory subunit 12C (*PPP1R12C*) gene.^66^ Follow-up studies using a genome-wide approach identified further recurrent integration sites at RBS on chromosome 5p13.3 (AAVS2) and chromosome 3p24.3 (AAVS3).^67^ These recurrent integration sites (AAVS1-AAV3) have also recently been seen in samples from humans and macaques.^68^

There are limitations to the extrapolation of data from wild-type AAV for rAAV vectors due to the removal of Rep sequences required for site-directed integration. A detailed description of the processes influencing molecular persistence with a focus on AAV integration for rAAV has recently been published.^69^ Our group systematically evaluated mechanisms of long-term persistence of rAAV after a 10-year follow-up in a hemophilia A canine model.^70^ Within this study, the predominant vector form was episomal structures with copies correlating with transgene expression. Additional findings in this study were of integration in all transduced liver samples at average frequencies of approximately 1 × 10^−3^, with some common integration sites associated with areas of open chromatin. Supporting primarily episomal-derived expression, long-read sequencing of higher-frequency integration sites only detected fragmented and rearranged integrated vector forms. Similar findings of recurrent integration after long-term follow-up have been seen in another hemophilia A dog model.^71^ However, a recent report of integrated concatemers in NHPs treated with AAV8 vectors encoding rhLDLR, hLDLR, or GFP transgenes suggests that further evaluation of the source of transgene expression is required.^72^

There are limited studies evaluating the mechanism of persistence in clinical trial participants to date.^73–77^ For a muscle-directed AAV1 vector (AAV1-LPL^S447X^) used for treatment of lipoprotein lipase deficiency biopsy samples demonstrated predominant episomal persistence with low integration frequencies (1 × 10^−4^ to 1 × 10^−5^).^75^ Studies evaluating liver-directed AAV for treatment of acute intermittent porphyria rAAV2/5-cohPBGD and hemophilia A (AAV5-HLP-cohFVIIISQ) have demonstrated episomal persistence within the liver with integration seen at low frequencies (1.17 × 10^−3^ and 3.97 × 10^−3^ respectively).^73,76,77^ Episomal persistence has been described for rAAV for a number of different vectors. Collectively, these studies indicate that despite the common belief that AAV is a nonintegrating virus, low frequencies of integration have been observed.

### Clinical trials: A brief overview of progress and challenges

**Figure 1.**
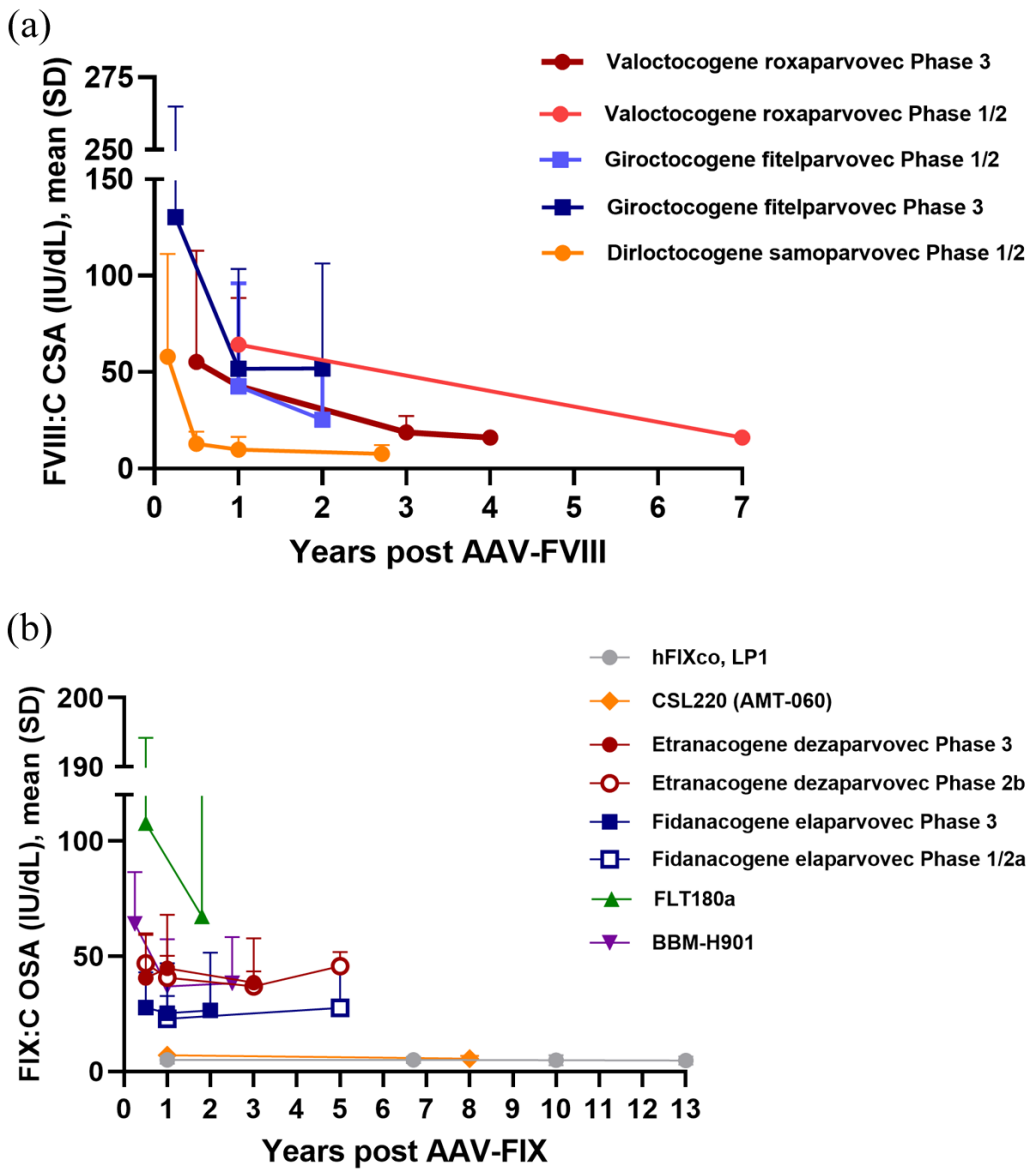
Variability and durability of approved and investigational FVIII and FIX gene therapies. (a) FVIII:C levels post-AAV-FVIII gene therapy. All data points represent mean, and standard deviation (when available) of CSA values.^18,21–29,32,33,85^ For dirloctocogene samoparvovec, values were estimated from Supplemental Figures; FVIII:C one-stage assay values were converted to chromogenic substrate values by a factor of 1.5; 2/18 participants in this study lost all FVIII:C and are not included in these results.^29^ For giroctocogene fitelparvovec phase I/II data, median FVIII:C (CSA) data are presented.^28^ For phase I/II clinical studies, plotted results are based on the following vector doses: valoctocogene roxaparvovec—6 × 10^13^ vg/kg (*n* = 7); giroctocogene fitelparvovec—3 × 10^13^ vg/kg (*n* = 5); dirloctocogene samoparvovec—2 × 10^12^ vg/kg (*n* = 7). (b) FIX:C levels post-AAV-FVIII gene therapy. All data points represent mean and standard deviation (when available) of OSA values.^13–15,37–49,84,86–88^ For phase I/II clinical studies, plotted results are based on the following vector doses: CSL220—2 × 10^13^ (*n* = 5); hFIXCo, LP1—2 × 10^12^ vg/kg (*n* = 6); verbrinacogene setparvovec—8.32 × 10^11^ vg/kg (*n* = 4). Notably, CSL220 and scAAV 2/8-LP1-hFIXco use the wild-type FIX, and all other gene therapies employ the FIX Padua construct. AAV, adeno-associated virus; CSA, chromogenic substrate assay; FVIII, factor VIII; FIX, factor IX; OSA, one-stage assay.

Early clinical trials of AAV-based hemophilia gene therapies involved intramuscular delivery and hepatic artery infusion of AAV2-FIX.^78–80^ These studies demonstrated the potential for sustained FIX expression and identified a T-cell–mediated immune response to AAV capsid antigens, resulting in loss of FIX-expressing hepatocytes.^79,81^ Subsequent administration of reactive immunosuppressive corticosteroids on detection of transaminase levels greater than twofold above baseline resulted in maintenance of FIX expression at levels of 5%–7% up to 13 years posttreatment (Figure 1(a)).^44,46,47,82–84^ More recent hemophilia B gene therapy programs using the FIX “Padua” variant to enhance FIX specific activity have shown sustained levels in the range of mild hemophilia, associated with a significant decrease in annualized bleeding rates (ABR), and improved health-related quality of life.^13–15^ In contrast, one phase I/II study (B-AMAZE) reported supraphysiological peak FIX levels (FIX:C >150 IU/dL) in patients treated at a dose of 8.32 × 10^11^ with a more marked decline in the first year after completion of prophylactic immune suppression compared to those treated at lower doses.^13^ In this study, decline in expression in some patients likely relates to an immune response seen on tapering of immunosuppression with occurrence of late transaminitis reported. Within a separate phase I/II study (BBM-H901), smaller declines were seen in the first year following an initial mean peak of 64.1–36.9 IU/dL at week 58 which remained stable at 2 year follow-up.^13,42,49^

The FIX gene therapy etranacogene dezaparvovec (AMT-061, Hemgenix^®^, CSL Behring, King of Prussia, PA, USA) is currently approved in the United States, Canada, United Kingdom, Australia, Korea, Taiwan, and Hong Kong and conditionally approved in Europe for the treatment of adults with severe and moderate hemophilia B. The efficacy and safety of etranacogene dezaparvovec were evaluated in a phase III clinical study involving 54 males with FIX:C ⩽2 IU/dL.^14,36^ FIX:C levels remained stable for up to 3 years posttreatment, with a mean FIX:C of 41.5% at year 1, and 38.6% at 3 years following treatment. Peak FIX:C levels seen in this study were not as high as those seen in the B-AMAZE and BBM-H901 studies with more stable FIX expression kinetics. Compared to a 6-month lead-in period of FIX prophylaxis, the mean ABR decreased from 4.1 to 1.51 with etranacogene dezaparvovec. Phase IIb data showed similar durability, with mean FIX:C levels of 40.8 IU/dL at year 1 and 45.7 IU/dL at year 5 in three adult participants.^38^ Two of the three participants remained bleed-free during the 5-year follow-up period.

A second FIX gene therapy (SPK-9001, Beqvez^®^, Pfizer, New York, NY, USA)^86^ was approved in the United States and Europe in 2024 but was withdrawn for commercial reasons. Additional FIX gene therapies are currently under clinical evaluation (Table 2). Recently, data from a 13-year follow-up of 10 males with severe hemophilia B who received scAAV2/8-LP1-hFIXco vector (2 × 10^11^ to 2 × 10^12^ vector genomes (vg)/kg) were described.^84^ FIX activity levels remained stable over time, the mean ABR decreased from 14.0 before gene therapy to 1.5, and no long-term safety concerns were reported.

Valoctocogene roxaparvovec (AAV5-FVIII-SQ, Roctavian^®^, BioMarin Pharmaceutical, San Rafael, CA, USA) was the first gene therapy approved in the United States and conditionally approved in Europe for the treatment of adults with severe hemophilia A. In phase I/II and III clinical trials, mean FVIII activity levels were 64.3% and 42.9%, respectively, by chromogenic substrate assay (CSA) 12-months posttreatment with a gradual decline to levels in the range of mild hemophilia over 2–7 years of follow-up (Figure 1(b)).^21,22,24^ Evaluation of phase I/II efficacy outcomes in the 6 × 10^13^ vg/kg cohort (*N* = 5) up to 7 years posttreatment showed a >88% decrease in ABR that was maintained despite a reduction in mean chromogenic FVIII activity to 16.2 IU/dL. The estimated rate of FVIII activity decline was −0.001 over year 7, interpreted as showing stabilization of FVIII expression.^24^ Overall, treatment was associated with significantly reduced ABR and exogenous factor use, and improved health-related quality of life.^22,89^ Similar expression profiles and reduction or loss of transgene expression have been observed in association with several other smaller FVIII and FIX AAV gene therapy studies, particularly in cases where initial expression levels are high, with decline occurring within the first year posttreatment.^23,29,40^

Despite these achievements, challenges still exist in the development and delivery of hemophilia gene therapy. Anti-AAV antibodies may impact efficacy, potentially limiting patient eligibility and restricting treatment to a single administration. Delayed cellular immune response to AAV capsid proteins can result in the loss of transgene-expressing hepatocytes, with some clinical trial participants refractory to corticosteroid treatment.^29^ Additionally, the mechanism of action of corticosteroids and optimal treatment regimen are not fully understood. Finally, the variability and durability of transgene expression, with an overall decline over time associated with FVIII gene therapy, pose significant challenges to achieving long-term therapeutic benefits and consistent outcomes for patients. Additional studies are needed to develop strategies to reduce immunogenicity and better understand the mechanisms underlying variability in efficacy and treatment durability.

## Species translatability of AAV gene therapy studies

Various animal models have provided important data on efficacy and safety of novel therapeutics for the treatment of hemophilia, each with different advantages, limitations, and ethical considerations. For the study of hemophilia, spontaneous mutations in the *F8* or *F9* genes have not been reported in mice, requiring the use of models with targeted gene disruption. The most widely used models have been induced through neo-cassette insertion into exon 16 or 17 of the *F8* gene or into exon 3 of the *F9* gene (reviewed in: Sabatino et al.^7^ and Yen et al.^90^). Transgenic knock-in mice expressing human FVIII or FIX with pathogenic variants (R593C-hFVIII and R333Q-hFIX) have also been described.^91,92^ Although these mice do not express endogenous FVIII or FIX, there are limitations for the evaluation of therapies with long-term implications, due to a lack of spontaneous bleeding phenotype and relatively short life span (1–2 years). Additionally, the use of vectors containing human *F8* or *F9* within the expression cassette has generally required immune-deficient animals, limiting the ability to evaluate immune responses in vivo.

Canine models of hemophilia have provided important insights into hemophilia therapeutics, including AAV. In contrast to mice, hemophilia A and B spontaneously occur in different breeds of dogs. For hemophilia A, a genetic variant similar to the intron 22 inversion,^93^ one of the most common causes of severe hemophilia A in humans,^94^ has been identified in the colonies maintained at Queens University (Canada) and University of North Carolina (United States).^95,96^ The characterization of the canine *F8* gene^97^ has allowed the development of recombinant canine FVIII for treatment of bleeding events and production of canine-specific AAV constructs for the evaluation of gene therapy. These animals display a spontaneous bleeding phenotype predominantly affecting the soft tissues, limbs, or joints, and require treatment approximately 3–4 times/year. Similarly, spontaneous *F9* variants have been described in dogs, including point mutations (missense/stop) or deletions. These canine models have allowed for the evaluation of long-term outcomes at the natural lifespan of these animals.^10,71,98^

A sheep model of hemophilia A has also been described with a spontaneous bleeding phenotype, resulting from a stop codon and frameshift in exon 14 of the ovine *F8* gene.^99^ Gene therapy studies in this model have predominantly focused on autologous transplantation of transduced mesenchymal stem cells.^100,101^ Finally, NHP models are used as a final stage of preclinical safety evaluation for many therapeutics, although in the setting of hemophilia, this is limited by the absence of spontaneous *F8* or *F9* pathogenic variants and the propensity for immune responses to human transgenic proteins.

Within these models, there are also limitations in the laboratory assessment of protein expression. For human studies, there are international plasma standards for both FVIII and FIX with validated assays comprising human-specific reagents. Murine and canine plasma standards do not exist, and these studies often rely on the creation of normal plasma pools, which are then used for the development of standard curves to assign activity. Although this allows for comparison to normal levels within a particular species, it provides a less well-defined standard and may be subject to differences in the transgene-produced protein. Interestingly, similar assay discrepancies have been reported between FVIII one-stage assay (OSA) and chromogenic activity assays (with OSA > CSA) in murine and canine models, as well as in human trials with B-domain deleted FVIII proteins. Higher OSA than CSA activity for AAV5-FVIII-SQ may be caused by accelerated early FXa formation compared with native FVIII in the human matrix,^102^ while matrix interference appears to mediate the differences in murine matrix (S. Fong, personal communication).

Despite these potential limitations, animal models have provided translationally relevant data on cellular mechanisms, efficacy, and safety of AAV gene therapy. Due to substantial differences in AAV tropism among species, it is essential to evaluate key factors influencing translatability to bridge the gap between preclinical findings and clinical outcomes. A consistent trend observed across mice, NHPs, and humans is the general pattern of peak high expression, gradual decline and stabilization in factor expression over time, with varying temporal kinetics.^11,18,29,32,72,85,103–105^ The expression pattern in hemophilia dogs appears to be lower and more stable over time. In the absence of anti-therapeutic protein antibodies, transcriptional regulation appears to be the primary mechanism driving this decline across species, albeit genome degradation and translational shutdown were also reported. Notably, while vector genome retention is relatively similar from rodents to large animals and primates, transcriptional efficiency sharply declines in higher species, including humans. Here, we summarize available data from preclinical and clinical studies of hemophilia B (wild-type FIX (CSL220/AMT-060) and FIX Padua (AMT-061) and hemophilia A (AAV5-FVIII-SQ (valoctocogene roxaparvovec) and AAV8-FVIII-V3) to identify patterns that may inform future therapeutic strategies.

### Cross-species analysis of AAV-FIX and AAV-FIX Padua translatability

Both wild-type AAV-FIX and AAV-FIX Padua gene therapies have been evaluated in preclinical murine, canine, and NHP models. For all species, AAV-FIX Padua has shown enhanced specific activity compared with wild-type AAV-FIX, consistent with the results of human clinical studies. In mouse models, therapeutic levels of FIX were achieved at lower doses than currently approved and investigational FIX gene therapies.^106,107^ Long-term durability was evaluated in a single cohort of mice (*n* = 6) treated with 4 × 10^11^ vg/kg AAV8-hFIX; mice exhibited supraphysiological peak hFIX levels of 300%–400% after 8 weeks which then plateaued to ~109% at 40 weeks posttreatment.^106^ This model showed a clear decline of FIX expression associated with high initial FIX expression, with the proportional reduction in activity amplified by the effect of the FIX Padua variant.

In hemophilia B dogs receiving 1.1–12 × 10^12^ vg/kg AAV2-cFIX, FIX activity was stable at 4%–10% for ~5.8 years,^98^ while hemophilia B dogs with and without preexisting anti-FIX inhibitors receiving 1–3 × 10^12^ vg/kg AAV2-cFIX Padua had peak FIX activity levels at 45%–325% and showed stable FIX expression in the 25%–325% range up to 3 years.^107^ Preclinical studies in cynomolgus monkeys compared the efficacy of CSL220 (AMT-060, AAV5-FIX) and AMT-061 (AAV5-FIX Padua, etranacogene dezaparvovec) for up to 26 weeks posttreatment.^108^ At the 5 × 10^12^ vg/kg dose used in clinical trials of AMT-060 and AMT-061, maximal FIX activity levels peaked at approximately 4 weeks postdosing (approximately 20% and 175%, respectively) followed by a gradual decline to plateau (approximately 7% and 50%, respectively) at 26 weeks posttreatment.^108^ Antibodies (inhibitors) against hFIX were detected after treatment with AMT-060 and AMT-061, regardless of the dose, and associated with a decline in hFIX expression.

### Cross-species analysis of AAV5-FVIII-SQ and AAV8-FVIII-V3 translatability

For AAV5-FVIII-SQ (valoctocogene roxaparvovec), FVIII peak expression levels were lower in both humans and NHPs compared to mice, despite relatively consistent liver vector genome copy numbers across species (Table 3).^74,103,109^ This discrepancy is attributed to reduced transcriptional efficiency in larger species. Similarly, preclinical data from AAV8-FVIII-V3 showed a comparable trend, where liver vector copy numbers were similar between mice and NHPs at equivalent doses, but FVIII expression levels in NHPs were 15- to 30-fold lower, reinforcing the impact of species-dependent transcriptional efficiency.^19^

**Table 3.** Cross-species evaluation of vector persistence and expression.

Species	Subject ID/sample size	Actual dose of AAV5-hFVIII-SQ (vg/kg)	Time after dosing (weeks)	Full-length DNA (copies/dg)	RNA/DNA ratio*	Peak FVIII, % normal (time)	FVIII, % normal corresponding to liver collection (CSA)
Mouse	N/A	2.9 × 10^13^	24	0.1	6366	65 (12 weeks)	67 (24 weeks)
		7.6 × 10^13^	24	1.3	11,857	380 (12 weeks)	233 (24 weeks)
Dog	*N* = 6	6 × 10^12^ to 2.7 × 10^13^	426–624	0.014–0.592**	69–731	6.5 (4.03–9.42) (99–619 weeks)	1.8–8.6
NHP	NA	6 × 10^13^	13	1.9	79	50 (3–5 weeks)	<LOD
		6 × 10^13^	26	3.2	140		
Human	1	6 × 10^12^	201	0.09	24	<LOD	<LOD
	11	4 × 10^13^	140	1.65	47	25 (52 weeks)	18.6
	5	4 × 10^13^	148	1.29	9	<LOD	< LOD
	3	6 × 10^13^	214	3.11	90	54 (28 weeks)	8.2
	4	6 × 10^13^	213	4.24	39	269 (21 weeks)	13.5

Source: Table adapted from previously described work.^10,26,74,109^

Notably, canine AAV-FVIII gene therapy studies were performed using noncodon optimized vector which may impact RNA levels. Overall, canine FVIII expression in responding dogs was stable. Peak canine FVIII level represents the highest chromogenic FVIII:C recorded across the study; variance in timing of peak compared to other species may be affected by differences in assay methodology in earlier compared to later study time points. Data are presented as a mean and range for six responding dogs and excludes results for two nonresponding dogs.

* RNA/DNA ratio: DNA is copies/ng DNA; RNA is copies/μg RNA.

** Overall vector genome levels (including full-length and truncated).

AAV, adeno-associated virus; CSA, chromogenic substrate assay; FVIII, factor VIII; LOD, limit of detection; NHP, nonhuman primate.

Further insights into translatability were derived from in vitro studies using primary hepatocytes from multiple species.^110^ These studies demonstrated that while vector DNA levels remained consistent across species, RNA levels were significantly lower in NHP and human hepatocytes compared to rodents. Importantly, NHP hepatocytes exhibited FVIII expression levels more closely resembling human hepatocytes than those of mice, supporting their relevance as a translational model.^111^ Additionally, a recent study by Kim et al.^112^ using machine-perfused human livers provided direct evidence that AAV transduction efficiency in human hepatocytes is best predicted by NHP models, as both species demonstrated similar capsid uptake and vector genome processing patterns. Mice with humanized livers in which primary human hepatocytes were transplanted into immunodeficient mice have been used to evaluate AAV-mediated transfer.^113^ They provide a platform to study human-specific hepatocyte responses in the whole organism. However, researchers must carefully consider experimental variables such as liver injury cycles and human donor variability to ensure the translatability of their findings.

Collectively, these findings highlight the critical role of transcriptional efficiency as a key determinant of AAV gene therapy expression across species. Understanding these differences will be essential in optimizing vector design, dosing strategies, and preclinical modeling to enhance clinical translation and therapeutic efficacy.

## Variability, durability, and strategies for enhancing gene therapy outcomes

Consistent and durable expression of the transgene is paramount to the success of AAV-based gene therapy for hemophilia A and B. However, variability in transgene expression across patients and within individuals presents significant challenges that must be understood and addressed. This section explores the factors influencing variability and durability, and potential strategies to optimize outcomes.

### Variability in AAV-based gene therapy for hemophilia: Mechanisms and implications

#### Clinical and preclinical observations of variability

Clinical studies of AAV gene therapy in hemophilia A and B have revealed substantial inter- and intraindividual variability in therapeutic response, with hemophilia A patients exhibiting greater variability (Tables 1 and 2). While some individuals failed to achieve factor expression, others have developed supraphysiological levels, leading to commencement of thromboprophylaxis.^114^ Additionally, factor expression fluctuated over time, influenced by liver health, immune responses, drug interactions, and other unknown factors.

This variability has also been observed in preclinical models, where mice, dogs, and NHPs exhibited significant interindividual differences in gene therapy outcomes.^10,103,115^ These models provide valuable insights into the mechanisms driving variability, allowing researchers to study AAV vector genome biodistribution/metabolism, immune responses, transcriptional regulation, and protein expression, all of which can help refine therapeutic strategies and improve clinical predictability.

#### Host-specific factors driving variability

Variability of transgene expression arises from a complex interplay of host-specific and nonhost mediated factors (Table 4, adapted from Pierce et al.^17^). Preexisting immunity to AAV capsids, particularly neutralizing antibodies, can reduce vector transduction efficiency although in one study this effect only appeared to be a factor at nAb titers >1 in 700.^14,116^ Moreover, host immune responses to the transgene product may contribute to progressive declines in expression.^117^

**Table 4.** Potential mechanisms mediating interindividual variability in responses to hemophilia AAV gene therapy.

Mechanism	Potential host-mediated mechanisms	Nonhost mediated mechanisms
Transduction	• Anti-AAV nAb or other plasma factors • Nonhepatocyte capture of AAV • Vector uptake • Uncoating • Repair/episome assembly • Retention of vector DNA	• Serotype • Vector dose • Manufacturing systems • Empty-to-full capsid ratios • Immune-suppression regimen
Transcript expression	• AAV transcription • Epigenetic regulation of vector genomes (methylation, histone interaction) • mRNA stability and clearance	• Promoter strength • Codon optimization • Drug-drug interaction
Protein production/function	• Translational regulation • Protein folding and secretion	• Variants with higher specific activity • Variants with better secretion
Post-transduction immunity	• Innate immunity • Adaptive immunity (cellular and humoral)	• Immune-suppression regimen
Other factors	• ABO group • von Willebrand factor levels • Clearance of FVIII/FIX • Underlying liver disease	• Hepatotoxic concomitant medications

Source: Adapted from Pierce et al.^17^

AAV, adeno-associated virus; FVIII, factor VIII; FIX, factor IX.

Beyond immune factors, genetic and epigenetic differences play a crucial role, as variations in transcriptional machinery and chromatin accessibility modulate transgene expression levels.^118,119^ Additionally, liver health and metabolic status influence AAV uptake and genome processing, further contributing to heterogeneity in clinical outcomes.^112,120,121^

Notably, endogenous FVIII levels exhibit significant interindividual variation, even among individuals with identical *F8* mutations in mild-to-moderate hemophilia A.^122^ A genome-wide association study (GWAS) analyzing over 32,000 individuals identified 7 new genetic loci influencing plasma FVIII levels, adding to ten previously reported loci associated with FVIII, von Willebrand factor (VWF), or both.^123^ Modifiers of FVIII clearance kinetics^124^ may contribute to variability in FVIII levels in the circulation, further highlighting the complexity of gene therapy outcomes.

#### Mechanistic insights from preclinical models

Studies using male mice administered AAV5-hFVIII-SQ (valoctocogene roxaparvovec) have provided further insights into host-driven variability.^104,126^ First, co-receptor expression was evaluated to determine correlations with vector transduction efficiency. Among the known AAV5 co-receptors, PDGFRα expression correlated with FVIII DNA levels, suggesting that PDGFRα may play a prominent role in AAV5 uptake.^[Bibr bibr125-20406207251406537] 126^ Beyond transduction, vector genome processing also contributed to variability. Artemis, a DNA repair enzyme involved in episome formation, correlated significantly with vector DNA levels, highlighting its potential role in determining the efficiency of vector processing and expression.^62,126^ Variability in FVIII transcript levels has also been significantly correlated with expression levels of the transcriptional regulators RNF121 (involved in AAV transcriptional elongation by RNA polymerase II), PHF5A (a pan-AAV transcription regulator), and HNF1α (a promoter-associated transcriptional regulator).^63,64,126^ These host factors likely modulate transgene expression and contribute to the observed heterogeneity in FVIII levels.

#### Clinical parallels: Insights from human liver biopsies

These preclinical findings align with observations in human liver biopsy samples collected from patients who received AAV5-hFVIII-SQ in a phase I/II clinical trial.^74^ Despite similar levels of vector transduction and the persistence of full-length circular episomal DNA, one patient exhibited low FVIII expression, classified as a nonresponder. This participant had reduced FVIII RNA transcript levels, along with downregulation of PHF5A expression compared to responders in the same study, mirroring findings in the mouse model.

Furthermore, genes involved in translation initiation and protein folding were positively correlated with FVIII expression.^74^ Interestingly, in three responders, higher plasma FVIII activity correlated with lower FVIII-SQ RNA levels, suggesting that cells may have a limited capacity for FVIII folding and secretion. The tendency of FVIII to misfold and aggregate remains a unique challenge in hemophilia A gene therapy.^127^ GRP78, a key ER chaperone protein, directly interacts with hFVIII-SQ, and variable GRP78 expression levels in human liver may contribute to differential transgene expression.^74,128^ Liver biopsy analysis showed plasma FVIII-SQ levels correlated with liver GRP78 RNA and protein expression, but there was no evidence of GRP78 overexpression in individual transduced hepatocytes expressing FVIII protein, ruling out ER stress at the time of sampling. To determine whether ER stress results from FVIII expression, research liver biopsies from humans are needed at the time of peak FVIII expression and/or during transaminitis. Further supporting the role of GRP78 in FVIII-SQ protein folding and secretion, a mouse study investigating AAV5-hFVIII-SQ driven by a stronger promoter than the one used in valoctocogene roxaparvovec at the dose of 6 × 10¹³ vg/kg demonstrated a significant increase in hepatic GRP78 expression.^129^ This modest upregulation positively correlated with plasma levels of FVIII-SQ protein, suggesting that GRP78 enhances hepatocyte capacity to fold and secrete FVIII-SQ. Additionally, increased expression of molecular chaperones such as PDIA5, PDIA6, HYOU1, and SDF2L1, components of the ATF6-mediated adaptive response to increase ER capacity, protein trafficking, and ER-associated degradation, were associated with higher FVIII-SQ levels. Notably, plasma GRP78 concentrations were also elevated and correlated with both hepatic GRP78 expression and circulating FVIII-SQ, indicating that plasma GRP78 could serve as a biomarker for predicting FVIII folding, secretion efficiency, and potentially ER stress. However, these observations require additional clinical validation. These findings align with human liver biopsy data, further emphasizing that interindividual variability in ER chaperone levels may contribute to differences in transgene expression and protein processing efficiency among patients.

#### Nonhost mediated factors affecting gene therapy response

Nonhost mediated factors, including vector design, manufacturing parameters, and immune-suppression regimens, play a critical role in variability observed in AAV-mediated gene transfer for hemophilia.^17^ Manufacturing-related differences can significantly influence the quality and performance of clinical vector lots. Variations in production platforms (e.g., HEK293 transient transfection vs producer cell lines), purification processes, and quality control of capsid populations affect the ratio of full to empty capsids, genome integrity, and levels of host cell contaminants. Each of these factors can influence initial transduction efficiency, the risk of immune activation, and the long-term stability of expression. Although direct clinical head-to-head comparisons are limited, these variables underscore the importance of robust analytical characterization and harmonized release criteria across programs.^130–132^ Differences in promoter strength can significantly impact transcriptional efficiency, as studies have shown that strong liver-specific promoters enhance transgene expression, while weaker promoters lead to reduced levels.^129,133^ However, in the context of FVIII gene therapy, using a stronger promoter may not be beneficial, as excessive expression can exceed the cell’s folding and secretion capacity, increasing the risk of intracellular stress, misfolding, and ER stress-related shutdown. Codon optimization has been shown to improve protein expression by aligning codon usage with host tRNA abundance, increasing translation efficiency.^134^ However, as noted in Faust et al.^49^ and Konkle et al.,^48^ certain optimization strategies may inadvertently increase CpG motifs and trigger innate immune responses (e.g., via TLR9). Therefore, while codon optimization improves expression, it may also introduce immunostimulatory signals relevant to long-term durability. Drug–drug interactions can also impact gene therapy outcomes, as seen in a clinical trial where isotretinoin treatment in a hemophilia A patient caused a sharp decline in FVIII expression, later confirmed in vitro to suppress FVIII mRNA levels.^110^ Similarly, efavirenz, an antiretroviral medication, has been shown to suppress AAV-derived RNA expression in vitro.^135^

Capsid type, vector dose, and capsid integrity also influence AAV uptake and transgene delivery, further contributing to inter- and intraindividual differences. Different AAV serotypes exhibit varying transduction efficiencies, with LK03 and AAV8 showing superior liver transduction compared to AAV2, affecting clinical efficacy.^136,137^ Vector dose plays a critical role in determining transgene expression levels, as higher vector doses result in increased expression but may also trigger immune responses and adverse events.^138^ Details of vector dosing need to be considered (i.e., vector particle numbers vs vector genome dosing), and integrity of the vector genome plays a key role in determining transgene expression efficacy. While there continues to be debate about the influence of the ratio of full to empty capsids, there is strong evidence that reducing the level of incomplete vector genomes in a preparation will benefit transgene expression.^139^

Immunosuppressive regimens are critical in modulating the variability of gene therapy outcomes, particularly in AAV-mediated treatments. The administration of prophylactic prednisolone has been explored to enhance transgene expression by mitigating immune responses. Preclinical studies in mice have demonstrated that prophylactic prednisolone enhances AAV5 hepatocyte transduction.^125^ The proposed mechanism involves upregulation of the AAV5 co-receptor PDGFRα, and suppression of innate immune responses, thereby facilitating increased transgene expression.

Similarly, studies in NHPs have shown that prednisolone administration reduces interferon responses to AAV vectors and may increase liver gene expression. In cynomolgus macaques, prednisolone treatment was associated with a decreased hepatic interferon gene signature and a trend toward higher vector genome and transgene expression levels, although these differences were not statistically significant.^140^ However, these effects appear to be species-specific. In a human phase IIIb clinical study, the use of prophylactic glucocorticoids, such as prednisolone, prior to administering valoctocogene roxaparvovec was associated with a decrease in transgene expression outcomes.^141^ This suggests that, in humans, prophylactic prednisolone may suppress necessary immune responses, leading to reduced therapeutic efficacy.^125,140^

These discrepancies highlight the complexity of immune modulation in AAV gene therapy across different species. While prednisolone may enhance transgene expression in animal models by modulating immune responses and receptor expression, its application in humans may lead to unintended suppression of transgene expression. Furthermore, clinical studies have demonstrated important differences between FIX and FVIII in the application of immunosuppressive therapy, with significantly less utilization in FIX versus FVIII studies. These outcomes indicate that careful patient surveillance during the first 6 months post-vector delivery, with selective short-term corticosteroid use, will eliminate harmful immunosuppressive side effects.

#### Durability challenges in AAV gene therapy for hemophilia

The key objective of gene therapy for hemophilia is to achieve sustained gene expression with a single administration. AAV-FIX gene therapy for hemophilia B has generally demonstrated durable transgene expression over multiple years, with minimal long-term decline at relatively low expression levels. In contrast, AAV-FVIII gene therapy for hemophilia A has shown greater variability and a more pronounced decline in expression across trials. These differences suggest that expression load may be a key determinant of durability, as higher FVIII expression levels have been linked to sharper declines. Supporting this, patients who achieve mild-to-moderate FVIII levels tend to maintain more stable expression over time, while those reaching supraphysiologic levels often experience a more rapid loss of expression. Notably, a similar pattern of early decline has also been observed in FIX gene therapy trials when initial expression levels were unusually high, further highlighting the impact of expression load across both transgenes (Figure 1). The development of AAV-based gene therapy for hemophilia A has been particularly challenging due to the large size of the *F8* gene, which complicates vector design and genome processing. Additionally, the FVIII protein, particularly B-domain deleted FVIII, is difficult to fold and secrete.^127^ Despite these challenges, preclinical and clinical studies across species including, mice, dogs, NHPs, and human liver biopsies have demonstrated long-term transgene expression, with vector DNA persisting primarily as circularized full-length episomes.^70,74,142,143^

Interestingly, hemophilia dogs exhibit durable FVIII expression following AAV gene therapy, with stability observed for more than 10 years.^10^ However, the lower expression levels seen in canine models compared to peak human clinical trial levels may contribute to their long-term stability by potentially reducing cellular stress and immune-mediated clearance. Understanding these species-specific differences provides critical insights for optimizing AAV-based gene therapies to achieve predictable and durable outcomes in humans.

In contrast to the canine model, declines in transgene expression have been observed in preclinical studies involving mice and NHPs.^72,103–105,144^ Sternberg et al.^144^ provided the first direct evidence that FVIII expression durability is dependent on expression load, demonstrating that higher peak FVIII levels correlated with a more pronounced decline over time in mice, a trend that mirrors human clinical trial data. The mechanisms contributing to these expression declines remain incompletely understood. Potential hypotheses include antibody-mediated clearance of the transgene product, loss of transduced hepatocytes due to cytotoxic T-cell (CTL) responses, cellular stress, hepatocyte turnover, host-mediated genome metabolism, and epigenetic silencing of vector genomes potentially triggered by ER stress-induced silencing or DNA repeat-mediated mechanisms.^17,145^ Additionally, the presence of unmethylated CpG motifs has been implicated in immune activation, contributing to loss of expression.^35^

Immune responses have been shown to play a critical role in the decline of transgene expression following AAV-mediated gene therapy. CTL responses targeting transduced hepatocytes have been observed to coincide with declining transgene expression, as evidenced in a clinical trial for hemophilia B, where a patient exhibited a decrease in FIX levels alongside elevated liver enzyme levels, indicating an immune-mediated attack on transduced cells.^79,81^ However, this temporal association between CTL activity and decline in expression is not consistently observed, even within the same trial or across different studies, suggesting additional factors may influence durability. Similarly, the development of antibodies against the transgene product can result in immune-mediated clearance and a subsequent decline in therapeutic efficacy. In some gene therapy studies, neutralizing antibodies against the introduced protein have been detected, leading to reduced transgene activity and limiting the therapeutic benefit.^146,147^ Of note, there have been no reported neutralizing antibodies (FVIII or FIX inhibitors) in hemophilia clinical studies reported to date.

A recent human liver biopsy study conducted as part of the phase III GENEr8-1 trial (NCT03370913) provided supportive evidence that transcriptional efficiency may play a role in the decline of FVIII expression following AAV gene therapy. In this sub-study, liver biopsies were collected from 12 participants between 0.5 and 4.1 years post-gene therapy administration.^143^ Histopathological analysis showed no signs of abnormal liver architecture or dysplasia, although some participants exhibited mild portal inflammation and variable levels of steatosis. Notably, transduction efficiency remained high across all biopsies, with over 88% of hepatocytes positive for vector genomes. Importantly, despite the stable presence of full-length episomal vector genomes over more than 4 years, two participants exhibited a sharp decline in FVIII activity, dropping from peak levels of 45.1 and 27.4 IU/dL at weeks 12 and 33, respectively, to below 3 IU/dL at biopsy collection (~weeks 138 and 170, respectively). These individuals had significantly lower FVIII-SQ RNA transcript levels, resulting in reduced RNA/DNA ratios, suggesting that the decline in FVIII expression was driven primarily by reduced transcriptional output rather than loss of vector genomes.

Further supporting the role of transcriptional regulation in the decline of transgene expression, a recent study in NHPs treated with AAV-FIX demonstrated that the decrease in FIX levels over time was linked to epigenetic modifications of the vector genome.^105^ The researchers observed a loss of active histone marks on AAV episomes, which correlated with a decrease in RNA production, despite the presence of similar vector genome copies in animals experiencing declines in expression. This suggests that epigenetic silencing mechanisms reduce transcriptional efficiency, contributing to the long-term decline in transgene expression, mirroring observations from human liver biopsy studies.

Similarly, a recent murine study demonstrated that the manufacturing system of AAV vectors can significantly influence epigenetic regulation and transgene durability.^104^ A comparison of AAV5 vectors produced in mammalian HEK293 cells and Sf insect cells revealed similar long-term expression patterns, but with distinct kinetics of expression. Vectors produced in HEK293 cells initially exhibited higher transgene expression, followed by a progressive decline that correlated with a reduction in vector genome copies in the liver, suggesting genome loss was a key factor in declining expression. Notably, HEK293-produced vectors elicited a heightened immune response compared to Sf-produced vectors, which may have contributed to the loss of vector genomes.^104^

Further supporting the role of vector genome loss in expression decline, Sternberg et al.^144^ demonstrated that AAV8 vectors produced in HEK293 cells exhibited a progressive reduction in vector genome copies over time, particularly in mice expressing high levels of FVIII protein. This trend suggests that higher antigen load contributes to accelerated vector clearance, potentially through an ER stress-mediated mechanism, as previously observed when FVIII was expressed under a strong promoter in conjunction with a high-efficiency vector such as AAV8 or with hydrodynamic delivery.^129,148–150^

In contrast, vectors produced in Sf cells initially exhibited lower levels of transgene expression but maintained stable vector genome levels over time. Despite this, transgene expression still declined, albeit through different mechanisms. Notably, this decline was associated with reduced transcriptional efficiency, likely due to epigenetic modifications affecting transcriptional accessibility, rather than vector genome loss.^104^ However, this pattern has not been consistently observed in the clinical setting. For example, in the phase III HOPE-B trial of the Sf9-produced etranacogene dezaparvovec, durable FIX expression has been demonstrated in long-term follow-up.^14,36,37,151^

A recent study in hemophilia A mice identified translational shutdown, where FVIII protein production decreased despite stable FVIII mRNA levels, contributed to the decline in FVIII expression.^152^ This effect was associated with ER stress and the activation of the unfolded protein response (UPR) in hepatocytes. Notably, interventions targeting interleukin-15 (IL-15) and the use of rapamycin alleviated ER stress and restored FVIII expression, suggesting potential therapeutic strategies to mitigate translational shutdown and improve transgene expression durability. These findings highlight the importance of addressing translational control mechanisms and ER stress responses to enhance the long-term efficacy of AAV-mediated gene therapies.

These observations suggest that excessive antigen production may contribute to loss of transduced hepatocytes, possibly due to ER stress-induced responses or immune-mediated clearance, highlighting the need for optimized expression levels to balance efficacy and durability. The clinical relevance of these findings remains uncertain, necessitating further research. Utilizing preclinical models alongside human liver biopsies from gene therapy recipients could provide critical insights into the mechanisms underlying FVIII expression loss and help guide strategies to improve long-term durability in hemophilia A gene therapy.

These findings collectively underscore the complexity of factors influencing transgene durability and suggest that transcriptional regulation, epigenetic modifications, vector manufacturing differences, genome loss, and translational efficiency all play critical roles in determining long-term expression levels following AAV gene therapy. Importantly, variability and durability are not separate challenges but rather interconnected aspects of transgene expression. Early variability in gene therapy outcomes, whether driven by immune responses, transcriptional regulation, or vector processing, can influence long-term durability, as the same mechanisms that cause fluctuations in initial expression levels may also contribute to progressive loss over time. For example, immune-mediated events (often manifesting as ALT elevations) can coincide with short-term decreases in factor levels in a subset of participants; however, across published datasets, including valoctocogene roxaparvovec, a consistent link between early transaminitis and a steeper long-term rate of decline has not been established.^24,153^ In the valoctocogene roxaparvovec program, ALT elevations were frequent and sometimes accompanied by ⩾30% contemporaneous reductions in FVIII activity, yet long-term declines have also been associated with reduced hepatocellular transcription (lower RNA/DNA ratios) despite persistent circular episomes, indicating additional nonimmune mechanisms can drive durability loss.^17,74,154^ Similarly, epigenetic modifications or stress-induced translational shutdown, initially responsible for interindividual differences in expression, may later dictate the extent of transgene expression persistence. Addressing variability at the outset may therefore be key to ensuring stable and durable transgene expression in the long term. While previous studies have highlighted potential immune-mediated mechanisms of expression loss, these data from human biopsies, NHPs, and murine models provide evidence that multiple mechanisms, including epigenetic modifications leading to decreased transcription efficiency and ER stress-induced genome loss (through hepatocyte death) or translational shutdown, contribute to declining factor levels over time. Further research into the molecular mechanisms regulating episomal transgene transcription, genome stability, ER stress response, and translational control is needed to optimize the durability of AAV-mediated gene therapy.

#### Future strategies to overcome variability and enhance durability in AAV gene therapy

Addressing variability and ensuring durable transgene expression in AAV gene therapy requires a multifaceted approach. Patient stratification currently involves prescreening individuals based on immune status (e.g., neutralizing antibodies) and liver health, and in the future may include genetic markers (e.g., *IL6R*).^34,155^ Immune modulation, through current use of corticosteroids or future use of newer immunosuppressants such as IL-15 inhibitors can mitigate immune-driven variability and prevent decline.^152^ Capsid engineering and transgene optimization can enhance transduction efficiency, transcriptional efficiency, genome persistence, and minimize immune recognition and potential UPR.^17^ Transcriptional and epigenetic regulation also play significant roles in gene expression consistency and durability; strategies such as histone deacetylase inhibitors,^115,156^ transcriptional enhancers, and codon optimization can help maximize transgene output. ER stress mitigation through small-molecule chaperones and protein-folding enhancers can address translational shutdown and improve FVIII secretion.^157^ Optimizing AAV vector manufacturing, ensuring high capsid integrity, reducing empty capsids, and minimizing contaminants, can further enhance therapeutic consistency. Avoidance of drugs implicated in the decline of expression, such as isotretinoin and efavirenz, is necessary.^110,135^

Re-dosing presents a major challenge in AAV gene therapy due to the development of high-titer anti-AAV neutralizing antibodies after initial administration. Emerging approaches to enable re-dosing include plasmapheresis, immune adsorption techniques, B-cell depletion therapies (e.g., anti-CD20 monoclonal antibodies), and CD19 CAR-T-targeted therapies to reduce preexisting antibodies, albeit their success is variable.^158^ Additionally, novel serotype switching strategies, where a second dose is delivered using a different AAV capsid with reduced cross-reactivity, may allow for successful re-administration. In support, a long-term follow-up of AAV-cFVIII-treated hemophilia A dogs found the predominant humoral response was toward the dosed capsid. Some cross-reactivity was observed toward other AAV capsids at early (<2 years) time points, with more limited cross-reactivity observed during the 2–12 years follow-up.^10^ Nonviral delivery methods are also being explored as potential solutions to extend the therapeutic window.^159^ A standardized method for evaluating anticapsid antibodies is also required. By integrating strategies for immune modulation, vector design (including capsid and transgene), transcriptional regulation, ER stress mitigation, and re-dosing, the field can progress toward a more predictable, durable, potentially titratable, and patient-tailored gene therapy approach.

## Lessons learned and future directions

For more than two decades, preclinical models of hemophilia have played critical roles in understanding the safety and efficacy of AAV-mediated gene therapy. In the important areas of assessing AAV transduction, transcriptional efficacy, and ER stress, access to longitudinal liver biopsies from mice, dogs, and NHPs has been extremely informative. During the introduction of novel strategies for gene transfer, these preclinical models are likely to be particularly valuable.

Many of the challenges to a successful gene transfer protocol relate to optimizing transgene delivery to the appropriate host cell type and to ensuring transgene DNA, mRNA, and protein levels are persistent over time. Objective and quantifiable evaluation of these metrics requires access to test organisms with similar anatomies, patterns of gene expression, and immune responses as those present in humans. There is long-standing evidence that these requirements are fulfilled in the range of mouse, dog, and NHP models available for detailed investigation. Furthermore, the ability to obtain longitudinal tissue biopsies in dog and NHP studies provides unique insights into gene therapy safety, efficacy, and mechanisms of action regulating gene therapy variability and durability of response, including the natural evolution of transgene persistence. The successful transition of preclinical approaches into the clinic in recent years has addressed key issues, including optimal vector dosing, immunogenicity, treatment during childhood, and vector re-administration, in an informative manner.

The next iterations of genetic therapies for hemophilia will likely involve two categories of innovation: (i) substitution strategies utilizing gain-of-function variants of FVIII in an attempt to overcome the decline in expression seen with current *F8* transgenes^144,160^ and (ii) various gene editing approaches that will in some instances require targeted delivery of the editing machinery (CRISPR-Cas9) to cell types that naturally express FVIII (endothelial subsets) or FIX (hepatocytes).^161,162^ The assessment of these approaches will again require preclinical evaluation to ensure efficacy and safety, with careful attention to inter-species variances.

## Conclusion

The availability of excellent animal models is one reason hemophilia has been a prime candidate for gene therapy. The ability to monitor safety and efficacy in detailed longitudinal studies has made the contributions of the murine and canine models invaluable for advancement into the clinic in recent years. Animal models have notably played important roles in assessing long-term safety issues associated with hemophilia therapeutics. These studies highlight the need to learn from animal models, particularly when evaluating therapeutics with irreversible outcomes such as gene therapy, to improve the safety profile of future treatments.

Preclinical investigations have provided us with critical knowledge about the durability of AAV-mediated therapy,^10,71^ immune responses to the transgenic protein,^163,164^ and the fate and form of AAV genomes many years after administration.^70,71^ Looking ahead, future novel genetic treatments for hemophilia are already being explored, and the integration of preclinical animal model studies will form an important part of the evaluative process. With this approach, not only do translational advances benefit human populations but also opportunities exist to give back to our long-term companion animals.^165^

## Declarations

**Ethics approval and consent to participate:** Not applicable.

**Consent for publication:** Not applicable.

**Availability of data and materials:** Data can be obtained from the corresponding author upon request.
